# Characterization of the complete chloroplast genome of *Allium prattii*

**DOI:** 10.1080/23802359.2018.1436994

**Published:** 2018-02-07

**Authors:** Fang-Yu Jin, Deng-Feng Xie, Song-Dong Zhou, Xing-Jin He

**Affiliations:** Country Key Laboratory of Bio-Resources and Eco-Environment of Ministry of Education, College of Life Sciences, Sichuan University, Chengdu, PR China

**Keywords:** *Allium prattii*, complete chloroplast genome, phylogenetic analysis

## Abstract

The genus *Allium* is one of the world’s largest monocot genera. However, few reports on the complete chloroplast genome of *Allium* plants are reported. In this study, we reported the complete chloroplast genome of *Allium prattii*. The genome sequence was 154,482 bp in length, including a large single copy region (LSC) of 83,392 bp and a small single copy region (SSC) of 18,064 bp, which were separated by two inverted repeat (IR) regions of 26,513 bp. The complete chloroplast genome contains 131 genes, including 85 protein-coding genes, 38 tRNA genes, and 8 rRNA genes. Phylogenetic analysis with several reported chloroplast genomes showed that *A. prattii* has a close genetic relationship with *A. cepa* and *A. sativum*.

The genus *Allium* comprises about 920 species (Seregin et al. 2015), making it one of the largest monocotyledonous genera. The *Allium prattii* C. H. Wright, a perennial herb, belongs to the family Amaryllidaceae, Sect. *Anguinum* (G. Don. ex W.D.J. Koch) (Herden et al. 2016; Li et al. 2016), and mainly concentrates on the Qinghai–Tibet Plateau and its adjacent regions. *Allium prattii* grows at an elevation from 2000 to 4900 m and adapts to very diverse habitats. In many areas, *A. prattii* is used as traditional Chinese medicine (Dahal et al. 2017) and important wild vegetable, as well as good pasture. Recently, few researches on the complete chloroplast genome of the genus *Allium* plants are reported. Knowledge of complete chloroplast (cp) genome would contribute greatly to our understanding of fair trade and harmony in the regulation of herbal medicines. We, here, assembled the complete cp genome of *A. prattii* to provide genomic and genetic sources for further research.

The mature and healthy leaves of a single individual of *A. prattii* was collected from Litang county (29°8′52.56″N, 100°4′10.74″E), Sichuan province, China, and were used for the total genomic DNA extraction with the modified CTAB method (Doyle 1987). The whole-genome sequencing was obtained 150 bp paired-end reads using the Illumina Hiseq Platform (Illumina, San Diego, CA). Adapters and low-quality reads were removed and high-quality reads were used for the cp genome assembly using Velvet version 1.2.10 (Zerbino and Birney 2008) and Oases version 0.2.09 (Schulz et al. 2012). The resulting contigs were linked based on overlapping regions after being aligned to *A. cepa* (KM088013) and visualized using Geneious version 11.0.4 (Kearse et al. 2012). Annotation was performed using Plann (Huang and Cronk 2015) which is a command-line application for annotating plastome sequences. A maximum-likelihood (ML) tree with 1000 bootstrap replicates was inferred using MEGA7.0 (Kumar et al. 2016) from alignments created by the MAFFT (Katoh et al. 2002) using plastid genomes of 10 species.

The complete cp genome of *A. prattii* (Genbank accession no. MG739457) was a circular molecular genome with a size of 154,482 bp in length, which presented a typical quadripartite structure containing two inverted repeat (IR) regions of 26,513 bp separated by the large single-copy (LSC) region of 83,392 bp and small single-copy (SSC) region of 18,064 bp. The cp genome consists of 131 genes including 85 protein-coding genes, 38 tRNA genes, and eight rRNA genes. The overall GC content is about 37.02%.

The phylogenetic analysis of 10 chloroplast genomes showed that *A. prattii* is closely related to *A. cepa* and *A. sativum* (Figure 1). This complete cp genome can be further used for population genomic studies, phylogenetic analyses, genetic engineering studies of *Allium*. Such genomic and genetic information would be fundamental to medicinal research.

**Figure 1.**
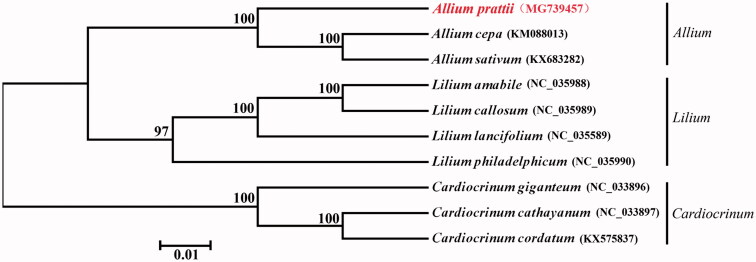
Phylogenetic relationship of *A. prattii* was constructed by chloroplast genomes with previously reported 9 species. Numbers in the nodes are the bootstrap values from 1000 replicates.
